# Supplementary material for: Differing Effects of Alcohol Use on Epigenetic and Brain Age in Adult Children of Parents with Alcohol Use Disorder
Source: Brain Sci. 2024 Dec 16;14(12):1263. doi: 10.3390/brainsci14121263 (PMC11674551; doi:10.3390/brainsci14121263)
Supplement: Supplementary file 1 [file brainsci-14-01263-s001.zip › brainsci-3341890-supplementary.pdf]

Supplementary Table S1: Assessment measures for adult children of a parent with alcohol use disorder (ACoA) based on current alcohol status, and control participants from unrelated studies representing a general population.

**Demographic Measure**		**Non-Hazardous**	**Hazardous**	**Control MRI**	**Control Genetic**
Gender (%)	Male	36	42	40	41
	Female	64	58	60	59
Age (range)		19-24	18-25	18-28	18-48
Race (%)	White	82	95	88	85
	African American	0	0	2	6
	Native American	0	5	1	0
	Asian American	5	0	6	9
	Multiracial	14	0	3	0
High School Class Size (%)	<50	20.00	28.57	*Unknown*	*Unknown*
	50-100	6.67	28.57	*Unknown*	*Unknown*
	100-150	20.00	14.29	*Unknown*	*Unknown*
	>150	53.33	28.57	*Unknown*	*Unknown*
Diagnosed/Treated Neurological or Psychiatric Disorder (%)		20.00	21.43	0	*Unknown*

**Assessment (Range)**	**Scale**	**Non-Hazardous**	**Hazardous**	**t Value**	**p Value**
CAST (0-30)		19.36±0.99	20.00±1.60	0.348	0.729
AUDIT (0-16+)		2.68±0.50	15.21±1.51	-8.337	*<0.001*
Duke Health Profile (0-100)	Physical Health	73.64±4.03	64.74±4.48	1.480	0.147
	Mental Health	72.73±5.06	52.11±5.05	2.868	*0.007*
	Social Health	71.36±4.43	54.74±5.43	2.397	*0.021*
	General Health	72.08±4.10	57.48±4.27	2.460	*0.018*
	Perceived Health	77.27±5.43	50.00±8.55	2.767	*0.009*
	Self Esteem	68.18±5.45	55.26±5.37	-1.677	0.102
	Anxiety	34.84±5.12	49.46±5.13	-2.006	0.052
	Depression	32.73±5.27	48.95±4.64	2.275	*0.029*
	Anxiety and Depression	32.15±4.90	51.76±5.44	-2.686	*0.011*
	Pain	29.55±6.29	47.37±8.09	1.762	0.086
	Disability	0.00±0.00	15.79±5.48	-3.108	*0.004*
BDI-II (0-63)		11.5±1.94	19.53±3.08	-2.270	*0.029*
BAI (0-63)		8.09±1.57	13.68±2.15	-2.136	*0.039*
PCL (17-85)		36.64±3.25	47.95±3.77	-2.286	*0.028*

MRI: Magnetic Resonance Imaging, CAST: Children of Alcoholics Screening Test; AUDIT: Alcohol Use Disorders Identification Test; BDI-II: Beck test for depression; *BAI:* Beck test for anxiety; *PCL:* PTSD Check List.
